# Effects of repeated balneotherapy on skin hydration and psychophysiological stress: findings from a 16-week korean spa trial

**DOI:** 10.1007/s00484-025-03034-y

**Published:** 2025-10-13

**Authors:** Hana Yu, Jinyoung Kwak, Sunhee Lee, Chang-Mok Lee, Jong-Min Woo

**Affiliations:** 1 Healthcare & Spa Industry Promotion Agency, 84, Yeomchi Sandan 1-gil, mbong-myeon, Asan-si, Chungcheongnam-do 31442 Republic of Korea; 2 Dept. of Sasang Constitutional Medicine, College of Korean Medicine, Daejeon University, Daejeon, 34520 Republic of Korea

**Keywords:** Balneotherapy, Skin hydration, Skin barrier function, Psychological stress, Community-based intervention

## Abstract

Balneotherapy has traditionally been associated with skin health and psychological well-being. However, few studies have assessed its effects during extended use in real-world, community-based settings. This study aimed to evaluate the effects of repeated balneotherapy on skin barrier function and psychological stress regulation in middle-aged women. A 16-week quasi-experimental trial was conducted in Asan, Republic of Korea, involving 58 community-dwelling women aged 40–64 years. Participants were assigned to either an intervention group (*n* = 29), which received biweekly 20-minute immersion sessions in naturally mineralized hot spring water, or a control group (*n* = 29) with no spa exposure. Primary outcomes included corneometry-based skin hydration and transepidermal water loss (TEWL). Secondary outcomes were assessed using the Stress Response Inventory (SRI), salivary cortisol, heart rate variability (HRV), and the Patient Global Impression of Change (PGIC). Significant in-group improvements were observed in the intervention group for skin hydration (*p* < 0.001), TEWL (*p* < 0.001), and SRI scores (*p* = 0.043). Between-group comparisons at week 16 showed significant differences for skin hydration, TEWL, SRI, and PGIC (all *p* < 0.05), whereas no significant differences were found for salivary cortisol or HRV. Repeated balneotherapy over 16 weeks improved skin barrier function and reduced psychological stress in middle-aged women. These findings support the feasibility of thermal bathing as a non-pharmacologic, community-based intervention for preventive wellness and highlight the need for larger randomized trials with long-term follow-up.

## Introduction

Balneotherapy—the therapeutic use of mineral-rich thermal spring water—has long been practiced in many parts of the world for its restorative effects on physical and psychological well-being (Fioravanti et al. 2011; Falagas et al. 2009; Matz et al. 2003). Defined by the World Health Organization and the European Spas Association as immersion in natural mineral waters for preventive or therapeutic purposes, balneotherapy is integrated into clinical and wellness systems in numerous countries (Gálvez et al. 2024). Its therapeutic potential spans a range of domains, including dermatology, musculoskeletal rehabilitation, and mental health.

Evidence for the dermatological benefits of balneotherapy is well established. Regular immersion in thermal mineral water has been shown to improve skin hydration, reduce transepidermal water loss (TEWL), and stabilize the epidermal barrier, largely due to the action of minerals such as magnesium, calcium, and bicarbonates on stratum corneum cohesion and lipid synthesis (Proksch et al. 2005; Jung et al. 2018; Huang et al. 2018; Kanwal et al. 2025; Moini Jazani et al. 2023). These effects are observed not only in patients with inflammatory skin conditions like psoriasis or atopic dermatitis (Jang et al. 2025; Protano et al. 2024) but also in healthy individuals, supporting its application in preventive dermatology.

Alongside skin health, balneotherapy has shown promise in modulating psychological stress. Previous studies have demonstrated reductions in stress-related symptoms and physiological markers—such as salivary cortisol and sympathetic nervous system activity—following thermal bathing (Rapolienė et al. 2015; Antonelli et al. 2024; García-López et al. 2024). In a notable early study, Toda et al. (2006) reported significant reductions in salivary cortisol levels after a single spa bathing session in healthy men, especially those with high perceived stress. Extending this evidence, Yang et al. (2018) reported that long-term balneotherapy significantly reduced mental stress and sleep disturbances in “sub-healthy” individuals, suggesting potential for holistic psychophysiological regulation through thermal immersion. These findings were supported by subsequent trials and reviews suggesting that thermal water exposure may facilitate autonomic regulation and vagal tone activation (Matzer et al. 2014; Gálvez et al. 2018; Lee et al. 2019; Saade-Lemus et al. 2025).

Despite being often studied separately, skin health and stress resilience are increasingly recognized as interconnected through shared neuroendocrine and immune mechanisms. Stress can impair skin barrier function by disrupting lipid synthesis, increasing permeability, and triggering inflammatory cascades (Arck et al. 2006; Elias 2007). Conversely, compromised skin integrity can contribute to psychosocial burden, reinforcing a vicious cycle of physiological and emotional dysregulation (Huang et al. 2018; Jang et al. 2025). This dynamic has been explored in the emerging field of psychodermatology and the concept of the skin–brain axis.

To reflect this interaction, the present study adopts a stress resilience framework, which emphasizes an individual’s ability to maintain or quickly recover homeostasis after exposure to stress. In this context, stress resilience was operationalized through a combination of subjective (Stress Response Inventory, SRI) and objective (salivary cortisol, heart rate variability [HRV]) indicators (Rapolienė et al. 2025; Frost et al. 2023; Saade-Lemus et al. 2025).

Although considerable research supports the independent effects of balneotherapy on either skin or psychological outcomes, relatively few studies have assessed both domains simultaneously—particularly in ecologically valid, community-based settings with prolonged exposure (Bender et al. 2014; Castelli et al. 2022). There remains a need to examine the feasibility and cumulative impact of repeated balneotherapy on multidimensional wellness outcomes in healthy populations.

Therefore, this study aimed to evaluate the effects of repeated balneotherapy on skin barrier function and psychophysiological stress regulation in middle-aged women in a real-world community setting.

## Materials and methods

### Study design and setting

This quasi-experimental study was conducted over 16 weeks (December 2021 to March 2022) in Asan, Republic of Korea. The study duration was selected based on prior balneotherapy research suggesting that cumulative effects on skin and stress outcomes emerge over multiple weeks (Matzer et al. 2014; Jung et al. 2018). Ethical approval was obtained from the Institutional Review Board of Daejeon University Cheonan Korean Medicine Hospital (IRB No. DJUMC-2021-10-003), and all participants provided written informed consent prior to enrollment.

### Participants

A total of 60 community-dwelling women aged 40–64 years were recruited via public advertisements and screened for eligibility by trained research staff.

- Inclusion criteria: generally healthy status confirmed by physician assessment; no dermatologic or psychiatric diagnoses; no participation in wellness or spa-related programs in the last three months.
- Exclusion criteria: pregnancy; chronic systemic diseases such as uncontrolled hypertension, cardiovascular disease, or autoimmune disorders; active infections; recent surgery; immunosuppressant therapy; skin conditions aggravated by immersion; and any contraindications for hot water immersion.

**Fig. 1:**
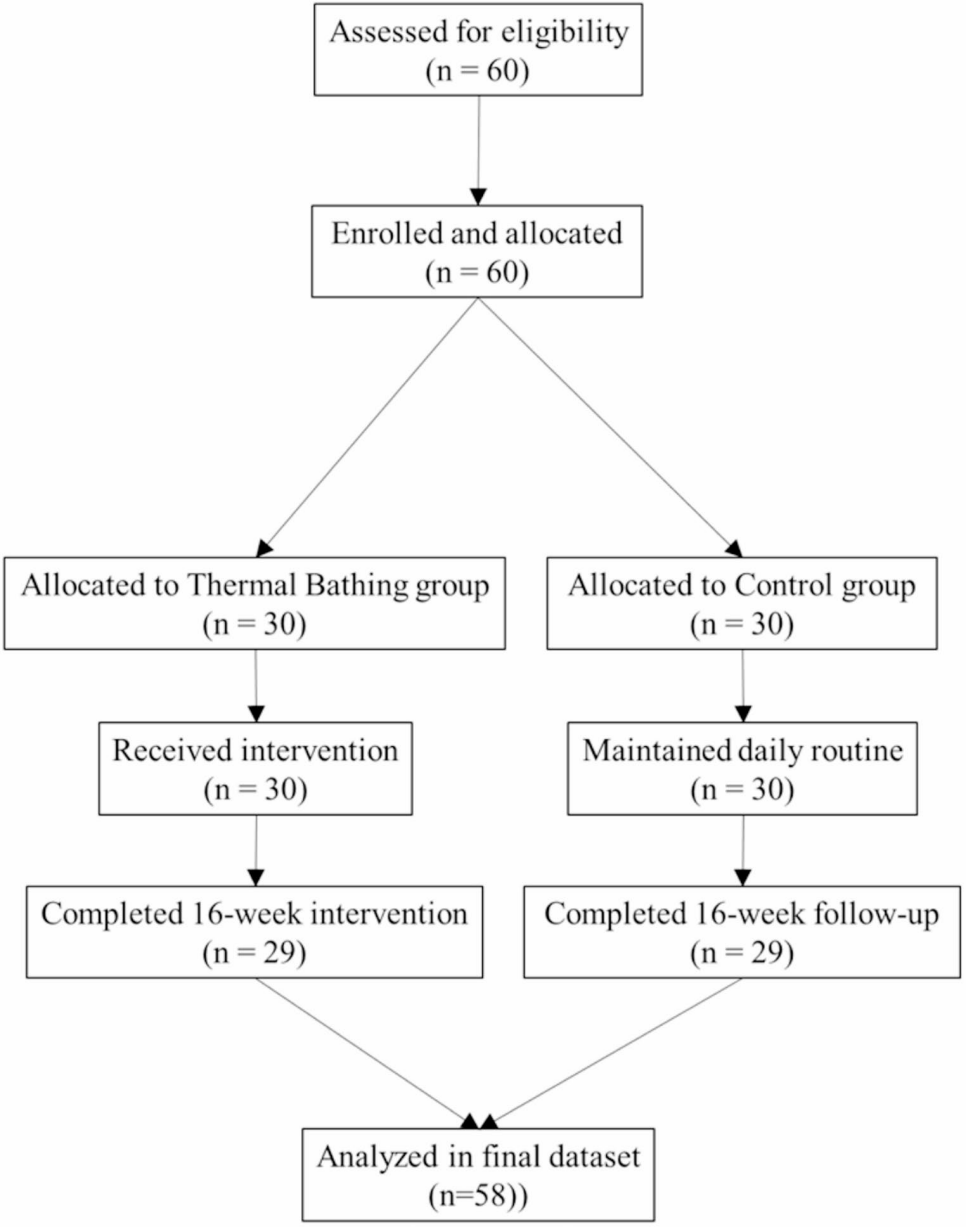
CONSORT Flow Diagram of Participant Progress Through the Study. A total of 60 women were assessed for eligibility. Two participants (one from each group) were excluded due to scheduling conflicts. The remaining 58 participants were assigned to the intervention group (*n* = 29) or control group (*n* = 29). All participants completed the 16-week assessment and were included in the final analysis

Information on daily routines was collected. The majority of participants were employed, while others were retired or homemakers. All participants were instructed to avoid additional spa bathing during the study and to maintain their usual hygiene and skincare routines. Participants were allocated to an intervention group (*n* = 30) or a non-intervention control group (*n* = 30), with 29 in each group completing the protocol (Fig. 1).

### Intervention protocol

The intervention group received biweekly (twice per month) full-body immersion sessions in Onyang hot spring water at the Shincheon Hot Spring Facility. Each session lasted 20 min in thermally regulated water maintained at 39–40 °C. The mineral composition of the hot spring water was as follows: pH 8.7, turbidity 0.14 NTU, chloride 34.7 mg/L, sodium 65.20 mg/L, silica 53.58 mg/L, and other trace elements as determined by facility analysis. Participants were instructed to avoid applying any skincare products on the day of immersion, and verbal reminders were provided before each session. Compliance was monitored by field coordinators.

### Outcome measures

#### Skin barrier function

Stratum corneum hydration was measured using a Corneometer CM825 (Courage + Khazaka, Germany). Transepidermal water loss (TEWL) was assessed using a Tewameter TM300.

#### Psychological stress and resilience

Subjective stress resilience was measured using the validated Korean version of the Stress Response Inventory (SRI), which includes emotional, somatic, and behavioral subscales. Global well-being was captured via the Patient Global Impression of Change (PGIC), a 7-point scale. Objective indicators included:

- Salivary cortisol: Collected via Salivette kits (Sarstedt, Germany) in the morning (08:00–09:00), following standard fasting and rest conditions. Samples were frozen at − 20 °C and batch-analyzed using ELISA.
- Heart Rate Variability (HRV): Measured using UBio Clip v40 (Biosense Creative, Korea) with participants in a seated, relaxed state for 5 min. Frequency-domain (LF, HF) and time-domain (SDNN) indices were collected.

### Data collection procedure

All measurements were conducted at baseline (week 0) and after the final session (week 16) under standardized indoor conditions (22–24 °C, 45–55% humidity). Assessors were blinded to group assignment.

### Statistical analysis

Data were analyzed using SPSS version 26.0 (IBM, Armonk, NY, USA). Normality was assessed via the Shapiro-Wilk test. Two-way repeated measures ANOVA was used to examine group × time interaction effects. Between-group comparisons of PGIC scores were conducted using independent t-tests. Missing data were handled using listwise deletion. Effect sizes were reported using partial eta-squared (η²), with thresholds for small (0.01), medium (0.06), and large (0.14) effects.

### Consort flow diagram

A CONSORT-style flow diagram illustrates participant enrollment, group allocation, retention, and analysis (Fig. 1).

### Results

#### Participant characteristics

A total of 58 participants completed the study and were included in the final analysis (29 in each group). Baseline comparisons revealed no statistically significant differences in demographic or physiological parameters. The mean age was 52.62 ± 6.49 years in the intervention group and 51.83 ± 7.13 years in the control group (*p* = 0.659). BMI was 23.9 ± 2.7 kg/m² in the intervention group and 23.8 ± 2.9 kg/m² in the control group (*p* = 0.912). Other baseline measures such as systolic blood pressure, diastolic blood pressure, resting heart rate, and body temperature were also statistically comparable (all *p* > 0.05), indicating appropriate group equivalence (Table 1).

**Table 1:** Baseline demographic and physiological characteristics of participants (*n* = 58)

Variable	Spa Group (*n* = 29)	Control Group (*n* = 29)	*p*-value
Age (years)	52.57 ± 6.53	51.83 ± 7.13	0.659
BMI (kg/m²)	23.9 ± 2.7	23.8 ± 2.9	0.912
Weight (kg)	61.23 ± 9.21	61.14 ± 10.67	0.957
Systolic BP (mmHg)	126.65 ± 10.88	124.68 ± 11.10	0.567
Diastolic BP (mmHg)	81.62 ± 8.55	79.96 ± 7.47	0.525
Resting Heart Rate (bpm)	70.96 ± 9.10	68.93 ± 7.80	0.435
Body Temperature (°C)	36.42 ± 0.35	36.22 ± 0.40	0.148

*BP* blood pressure, *bpm* beats per minute

#### Skin barrier outcomes

A significant group × time interaction effect was observed for skin hydration (F = 28.619, *p* < 0.001, η² = 0.339).

- In-group effects: The intervention group showed a significant increase in hydration from 39.08 ± 9.36 AU to 46.18 ± 10.08 AU (*p* < 0.001). The control group showed a decline from 44.15 ± 8.75 AU to 37.51 ± 7.42 AU (*p* < 0.001).
- Between-group effects: At week 16, hydration was significantly higher in the intervention group (*p* < 0.001).

For TEWL, a significant group × time interaction was also detected (F = 19.110, *p* < 0.001, η² = 0.255).

- In-group effects: The intervention group showed a reduction from 7.93 ± 2.18 to 5.55 ± 1.59 g/m²/h (*p* < 0.001). The control group increased from 6.45 ± 2.24 to 7.16 ± 2.61 g/m²/h (*p* = 0.038).
- Between-group effects: At week 16, TEWL was significantly lower in the intervention group (*p* < 0.001) (Table 2).

**Table 2:** Changes in skin hydration and transepidermal water loss (TEWL)

Variable	Pre (Spa)	Post (Spa)	Pre (Control)	Post (Control)	Group × Time Interaction (F, *p*)	Effect Size (η²)
Skin Hydration (AU)	39.08 ± 9.36	46.18 ± 10.08	44.15 ± 8.75	37.51 ± 7.42	F = 28.619, *p* < 0.001	0.339
TEWL (g/m²/h)	7.93 ± 2.18	5.55 ± 1.59	6.45 ± 2.24	7.16 ± 2.61	F = 19.110, *p* < 0.001	0.255

*TEWL* transepidermal water loss, *AU* arbitrary units

#### Psychological stress indicators

The SRI revealed a significant group × time interaction (F(1,56) = 4.285, *p* = 0.043, η² = 0.071).

- In-group effects: Scores decreased in the intervention group from 27.34 ± 18.97 to 20.62 ± 16.03 (*p* = 0.048), while remaining unchanged in the control group (22.62 ± 23.64 to 22.62 ± 21.04, *p* = 1.000).
- Between-group effects: At week 16, SRI scores were significantly lower in the intervention group (*p* < 0.05).(Table 3).

**Table 3:** Psychological outcome measures before and after the intervention

Variable	Pre (Spa)	Post (Spa)	Pre (Control)	Post (Control)	Group × Time Interaction (F, *p*)
SRI	27.34 ± 18.97	20.62 ± 16.03	22.62 ± 23.64	22.62 ± 21.04	F = 4.285, *p* = 0.043
SDNN (ms)	42.42 ± 20.42	34.94 ± 24.73	40.33 ± 9.25	37.36 ± 9.63	F = 0.735, *p* = 0.396
Stress Resistance	104.03 ± 14.60	96.72 ± 12.99	102.89 ± 7.06	100.10 ± 8.79	F = 1.642, *p* = 0.205
Salivary Cortisol (nmol/L)	90.41 ± 11.48	97.03 ± 10.84	90.10 ± 8.12	93.27 ± 8.59	F = 1.158, *p* = 0.286

*SRI* Stress Response Inventory, *SDNN* standard deviation of NN intervals, Stress Resistance, HRV-derived composite score; Cortisol measured from saliva samples

**Fig. 2:**
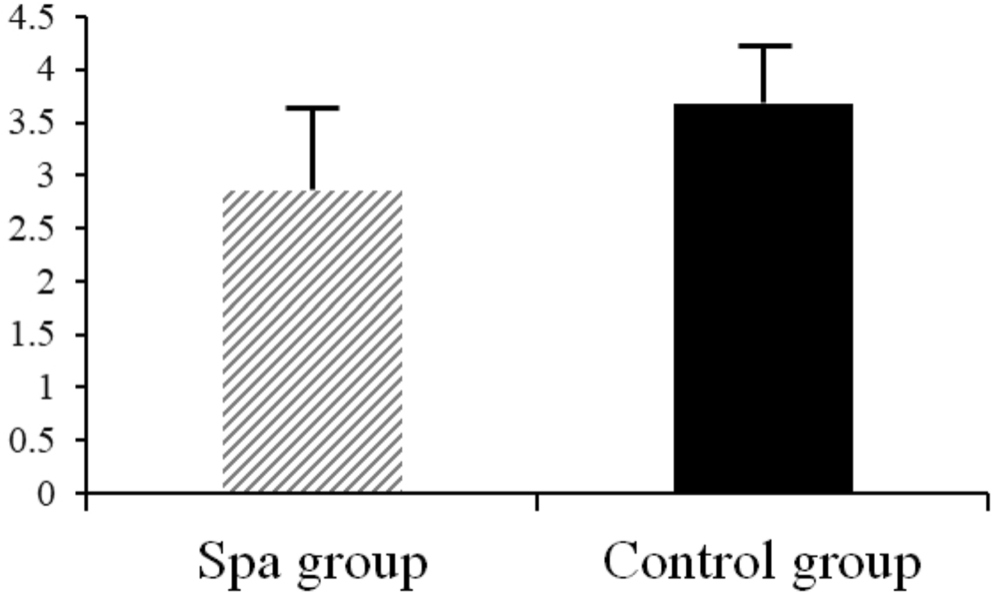
Patient Global Impression of Change (PGIC) scores at week 16 for the spa and control groups. The spa group reported significantly better perceived improvement (mean = 2.86 ± 0.78) compared to the control group (mean = 3.68 ± 0.54). Lower scores reflect greater perceived benefit (1 = “very much improved”; 7 = “very much worse”). Error bars represent standard deviations

PGIC scores also indicated superior perceived improvement in the intervention group (mean = 2.86 ± 0.78) compared to the control group (3.68 ± 0.54), with a significant between-group difference (t = − 4.656, *p* < 0.001) (Fig. 2). Lower scores indicate greater perceived benefit.

No significant group × time interactions were found for salivary cortisol (F(1,56) = 1.158, *p* = 0.286), SDNN (F(1,56) = 0.735, *p* = 0.396), or stress resistance (F(1,56) = 1.642, *p* = 0.205).

#### Correlation analysis

Pearson correlation analyses indicated no significant associations between changes in SRI and salivary cortisol (*r* = − 0.12, *p* = 0.34) or HRV indices (SDNN: *r* = − 0.09, *p* = 0.42). These results suggest limited concordance between subjective and biological stress markers.

#### Integrated summary of outcomes

To facilitate interpretation, Table 4 provides an integrated overview of both in-group and between-group results for all primary and secondary outcomes, including effect sizes.

**Table 4:** Integrated outcomes summary

Outcome	In-group effect (Intervention)	In-group effect (Control)	Between-group at Week 16	Effect size (η²/Cohen’s d)
Skin Hydration	↑ significant (*p* < 0.001)	↓ significant (*p* < 0.001)	Higher in intervention (*p* < 0.001)	η² = 0.339
TEWL	↓ significant (*p* < 0.001)	↑ significant (*p* = 0.038)	Lower in intervention (*p* < 0.001)	η² = 0.255
SRI	↓ significant (*p* = 0.048)	No change	Lower in intervention (*p* < 0.05)	η² = 0.071
PGIC	-	-	Better in intervention (*p* < 0.001)	d ≈ 0.85
Cortisol	NS	NS	NS	-
HRV (SDNN, Stress Resistance)	NS	NS	NS	-

↑ increase; ↓ decrease; *NS* not significant, *TEWL* transepidermal water loss, *SRI* Stress Response Inventory, *PGIC* Patient Global Impression of Change

## Discussion

This 16-week quasi-experimental study provides robust evidence that regular balneotherapy can yield significant benefits in both skin physiology and perceived psychological stress among healthy, middle-aged women. The observed improvements in skin barrier function were demonstrated by increased corneometric hydration and reduced transepidermal water loss (TEWL), supporting previous findings on the efficacy of mineral-rich thermal water in restoring epidermal moisture balance and lipid matrix integrity (Proksch et al. 2005; Jung et al. 2018). These results are clinically meaningful, as the magnitude of hydration improvement exceeded thresholds commonly applied in dermatological and cosmetic studies.

The concurrent improvement in perceived stress, as measured by the Stress Response Inventory (SRI) and the Patient Global Impression of Change (PGIC), further suggests that balneotherapy may serve as a dual-modality intervention addressing both physical and psychological dimensions of wellness. Although no significant changes were observed for salivary cortisol or heart rate variability (HRV), these null findings may reflect high inter-individual variability in physiological stress markers and the relatively low intervention frequency. Previous studies indicate that longer or more frequent exposures may be required to elicit measurable autonomic changes (Rapolienė et al. 2025).

While earlier research has demonstrated the effects of balneotherapy on skin or stress outcomes independently, relatively few studies have examined both domains simultaneously in ecologically valid, community-based settings. Our study contributes to this body of work by integrating dermatological and psychological endpoints within a 16-week protocol, targeting middle-aged women in Korea, and combining subjective and objective assessments. This design enhances ecological validity and reflects real-world preventive health applications beyond specialized spa clinics.

The divergence between subjective and objective stress indicators observed here also warrants attention. Psychological improvements were consistently reported, whereas biological markers did not show significant changes. This discrepancy may suggest that perceived stress relief is more sensitive to thermal bathing under the current intervention parameters, or that psychophysiological pathways operate with different thresholds of responsiveness. Future studies should consider stratifying participants by baseline stress levels, employing more frequent exposures, and incorporating ecological momentary assessments or wearable devices to capture dynamic physiological changes.

### Limitations

This study has several limitations. The quasi-experimental, non-randomized design restricts causal inference. The modest sample size, while adequate for primary outcomes, may have limited the detection of subtle changes in physiological stress markers. The absence of an active control group also makes it difficult to rule out placebo or expectancy effects. In addition, no post-intervention follow-up was performed, leaving the durability of observed effects uncertain. The trial was not registered on ClinicalTrials.gov, as it was classified as a non-randomized, community-based study, which may limit transparency and comparability with other clinical trials. Seasonal factors should also be considered, as the intervention was implemented during winter when transepidermal water loss (TEWL) levels tend to be elevated. Finally, reliance on self-reported stress outcomes introduces the possibility of expectancy or reporting biases.

Despite these limitations, the study provides important preliminary insights. By demonstrating simultaneous improvements in skin barrier function and subjective stress resilience under community-based conditions, our findings reinforce the potential of balneotherapy as a practical, non-pharmacologic wellness strategy. Future trials with randomized designs, larger and more diverse populations, and extended follow-up will be critical to validate these findings and to refine optimal treatment frequency and duration.

## Conclusions

Repeated balneotherapy over a 16-week period improved skin barrier function and reduced psychological stress in middle-aged women under community-based conditions. These findings support the feasibility of thermal bathing as a practical, non-pharmacologic wellness strategy. While objective markers such as cortisol and HRV did not significantly change, the overall pattern highlights the value of integrating balneotherapy into preventive health programs. Future randomized trials with larger and more diverse populations and extended follow-up are needed to confirm these results and to establish optimal treatment protocols.

## Data Availability

The datasets generated and analyzed during the current study are available from the corresponding author upon reasonable request.

## Abbreviations

AU: arbitrary units
HRV: heart rate variability
PGIC: Patient Global Impression of Change
SD: standard deviation
SDNN: standard deviation of NN intervals
SRI: Stress Response Inventory
TEWL: transepidermal water loss
